# A qualitative study on community use of antibiotics in Kinshasa, Democratic Republic of Congo

**DOI:** 10.1371/journal.pone.0267544

**Published:** 2022-04-27

**Authors:** Aurélie Koho Pungu Shembo, Patou Masika Musumari, Kriengkrai Srithanaviboonchai, Arunrat Tangmunkongvorakul, Olivia Dalleur

**Affiliations:** 1 Ecole de Santé Publique, Université Catholique de Louvain UCLouvain, Brussels, Belgium; 2 Global Health Interdisciplinary Unit, Center for the Promotion of Interdisciplinary Education and Research, Kyoto University, Sakyo-ku, Kyoto, Japan; 3 Research Institute for Health Sciences, Chiang Mai University, Sriphum, Muang Chiang Mai, Thailand; 4 Department of Community Medicine, Faculty of Medicine, Chiang Mai University, Sriphum, Muang, Chiang Mai, Thailand; 5 Clinical Pharmacy–Louvain Drug Research Institute, Université Catholique de Louvain UCLouvain, Brussels, Belgium; Rabin Medical Center, Beilinson Hospital, ISRAEL

## Abstract

**Background:**

Resistance to antibiotics is an increasing and major threat to global health. While the large majority of antimicrobial use occurs in the community where antibiotics are available without prescription, we did not find any studies investigating community-level factors influencing the inappropriate use of antibiotics in the Democratic Republic of Congo (DRC), where non-prescription antibiotic use is prevalent.

**Methods:**

This qualitative study was conducted from April 1^st^ 2019 to May 5^th^ 2019 and consisting of in-depth semi-structured interviews, utilizing purposive and snowball sampling schemes to recruit adult heads of households in the Pakadjuma slum, in Kinshasa, DRC. Participants with differing medical and educational backgrounds were selected. We employed a thematic analysis approach to explore community knowledge and use of antibiotics in the sampled population.

**Results:**

A total of 18 participants with a median age of 35 years were interviewed. The majority was female (77.7%), had at least a secondary education (83.4%), and unemployed (61.1%). We found that participants were familiar with the term “antibiotics”, but had limited knowledge of the indications and risks of antibiotics, including the risk of antibiotic resistance. Inappropriate use of antibiotics was common and there was frequent self-medication of non-prescribed medicines for a range of non-indicated conditions such as menstruation. Having limited income was the most commonly reported reason for not visiting a health facility for appropriate health care.

**Conclusion:**

Inappropriate use of antibiotics is a widespread practice and is influenced by lack of adequate knowledge of antibiotic use, indications and risks, prevalent self-medication, and financial barriers to accessing appropriate health care. There is need for both community education as well as structural interventions addressing poverty in order to reduce the inappropriate use of antibiotics in the Pakadjuma slum in Kinshasa.

## Introduction

Antimicrobial resistance is recognized as one of the significant global health challenges of the 21^st^ century, accounting for more than 700,000 deaths per year worldwide [1]. By 2050, antimicrobial resistance will cost approximately 10 million lives and about US$100 trillion if appropriate measures are not taken to halt its progress [1].

Antimicrobial resistance is included as a sustainable development goal [2, 3], and in many global initiatives by the World Health Organization (WHO) to improve the surveillance of antimicrobial resistance. These include for example the WHO tripartite database WHONET [4], the Advisory Group on Integrated Surveillance of Antimicrobial Resistance (AGISAR) and the Global Antimicrobial Resistance Surveillance System (GLASS) [5].

The inappropriate use of antimicrobials, including self-medication, sub-optimal dosage, and overuse of antibiotics, is considered a major driver of the emergence and spread of antimicrobial resistance [6–8]. Antibiotic resistance is a subset of antimicrobial resistance, and one of the important causes is the inappropriate use of antimicrobial agents, commonly called antibiotics. The inappropriate use of antibiotics depends on both supply and demand factors. Supply factors include the lack of antibiotic regulation, excessive prescription, and uncontrolled or uneven access to antimicrobials [9–11], while demand factors are linked to lack of consumer knowledge about appropriate antibiotic use and its implications, as well as beliefs, expectations and personal experiences with antibiotics [12–16]. Many studies suggest that the majority of antimicrobial use occurs in the community, where such drugs are readily available and can be obtained even without a prescription [17, 18]. A recent study estimated that community antibacterial consumption comprised approximately 85% to 95% of total antibacterial consumption in all nations for which data were available [19]. This means that patients themselves manage their antibiotics at home. They alone decide whether or not to follow the treatment, what to do with the leftover medicines, and how to store or dispose of them. A recent survey in Ghana found that 86.6% of antibiotics were used inappropriately, either because they were obtained without prescription or the full course of treatment was not completed [20]. Increasing evidence indicate that antimicrobial resistance in low-income countries occurs in micro-organisms that are likely to be transmitted in the community (e.g. such as pathogens causing pneumonia, diarrheal diseases, tuberculosis, sexually transmitted diseases, and malaria) [21].

In 1933, the Democratic Republic of Congo (DRC) passed legislation regulating the prescription and sale of pharmaceutical products. The law only allows qualified health personnel such as medical doctors to prescribe antibiotics and prohibits the sale of antibiotics by non-pharmacists [22]. Despite this, over-the-counter sales of antibiotics remain a common practice and are often handled by unqualified personnel. Antibiotics are highly accessible in retail drug shops (commonly called “pharmacies”) or even through street vendors [22]. Over-the-counter access to antibiotics including large-spectrum antibiotics poses a challenge in controlling inappropriate use of antibiotics and increases the potential risk of antibiotic resistance in the DRC and many other countries in Sub-Saharan Africa (SSA). In addition, access to health care in DRC is largely financed out-of-pocket (90% of the health care expenditure) in both the public and private health sector [23]. This in turn encourages self-medication practices as a way to avoid the cost associated with health care [22]. There is a need for research on community-level factors that are likely to impact access, quality, and use of antibiotics. Information regarding the community use of antibiotics in resource-constrained settings such as in Sub-Saharan Africa (SSA) remains remarkably scarce and there is no record of studies of this kind in the DRC.

The present study was designed to address this gap in the literature and aimed to explore community knowledge of antibiotics and practices in terms of use and storage of antibiotics among community residents of an urban slum in Kinshasa, DRC.

## Methods

This was a qualitative study conducted from April 1^st^ 2019 to May 5^th^ 2019 in the Pakadjuma slum, in Kinshasa, DRC. The Pakadjuma slum is located in Limeté township, one of twenty-four townships in Kinshasa. To date, there are no official reports or published studies describing the social structure and health care facilities in Pakadjuma slum. The slum is well-known for its lack of access to clean water and proper sanitation, making it one of the epicenters of the cholera epidemic in the DRC [24]. Commercial sex work is prevalent, which possibly contributes to the spread of sexually transmitted infections among its population. We purposively selected Pakadjuma slum as the setting of the study assuming a high prevalence of infectious diseases and antibiotic use among its population. The study population were adult (aged at least 18 years) heads of households with the power to decide on health-related issues for the household. The participants were purposely recruited using snowball and maximum variation sampling strategies. Maximum variation sampling was employed to ensure that a wide range of possible factors relevant to the study objectives were represented in the study population. These factors included level of education, employment status, household size, and medical background.

Data were collected through in-depth interviews, using a semi-structured interview guide. The interview guide was developed by the authors specifically for this study based on a scientific literature review on PubMed, cairn, and *Google Scholar*. The interview guide was assessed by the research team to ensure that it explored topics related to knowledge of antibiotics (indication of antibiotics, risks of antibiotics), methods of procurement, antibiotic sources, and practices related to the use and storage of antibiotics (including leftover drug) in the household. The interview guide was continuously modified throughout the study based on emerging themes from the interviews. The interviews (one interview per participant) were conducted in participant homes and lasted on average 45 minutes. The interviews were conducted in Lingala, the most commonly used language in Kinshasa. Researchers also conducted a visual inspection of medicines and their storage conditions in the household with photographic documentation.

All the interviews were digitally recorded with a smartphone, transcribed verbatim, and translated into French. All transcripts were reviewed for accuracy through comparison with the recordings. We used a thematic analysis approach to analyze the data. As described by Braun & Clarke (2006) [25], this analytical approach involves getting familiarized with the data through an iterative process of reading the transcripts, generating initial codes, arranging codes into larger categories, and drawing connections between codes and categories until a saturated thematic map of the analysis is generated [25]. The initial data coding was conducted by the principal investigator (AKPS), and the emerging themes were revised and refined through regular meetings with research team members PMM in the first phase and OD in the second phase. Discrepancies in coding were discussed and resolved by consensus and codes were organized into larger categories. For the purpose of this paper, participant quotes were translated into English to illustrate identified themes and were lightly edited for ease of reading.

### Ethics

This study was granted ethical approval from the National Health Ethics Committee, Ministry of Health, Kinshasa, DRC. (N 109/CNES/BN/PMMF/2018 of 8/04/2019). We obtained written informed consent from all participants prior to conducting the interviews.

All participants were informed of the study objectives, confidentiality, their right to choose to answer or not answer any question, and their right withdraw from the study freely at any time without any consequence or disadvantage. Eight potential participants declined to sign the written informed consents and were not included in the study.

## Results

### Description of participants

A total of 18 participants were interviewed. The median age of participants was 35 years. The majority were female (77.7%), had at least secondary education level (83.4%), and were unemployed (61.1%). Four of the participants had a medical background (3 nurses and 1 pharmacist) (Table 1).

**Table 1:** Sample characteristics.

Variable	N (18)	(%)
**Age**		
Median (Min-Max)	35	33–50
**Gender**		
Male	4	22.2
Female	14	77.7
**Education level**		
Primary	3	16.6
Secondary	6	33.3
High school/Professional training	5	27.7
University	4	22.2
**Profession**		
Unemployed	11	61.1
Employed	7	38.9
**Household size**		
Median (Min-Max)	4.5	3–10

We present results based on the major themes identified, namely, (i) Knowledge, indications, and use of antibiotics, (ii) Prescription of antibiotics, (iii) Dosage and duration of treatment, (iv) Storage of antibiotics in the household, (v) Antibiotic risks, (vi) Perceived quality of antibiotics available in the market, and (vii) Unreliable sources of information.

### Knowledge, indications, and use of antibiotics

All participants were familiar with the term “antibiotics” and most described antibiotics as drugs that were used against infections. Participants were able to give examples of antibiotics (amoxicillin, ampicillin, clamoxyl, ciprofloxacin etc.), though a few participants listed other drugs that were not antibiotics. All participants reported having personally taken or administered antibiotics to a member of the household.

“*Antibiotics are drugs… drugs that treat diseases*… *for example amoxicillins are antibiotics*”

(Female respondent #6)

The participants cited a range of signs and symptoms of infection for which they used antibiotics. These included fever; itching and pimples; cough; profuse sweating; ringworm; malaria (in combination with antimalarial drugs); and change in urine color and pain during urination. Several participants explained that the widespread use of antibiotics was the result of their unsanitary living conditions which exposed them to various types of infections. For example, several households shared latrines and bathing buckets, and the community at large lacked access to clean water.

“*Just looking at the environment we live in*, *you have to know that there are a lot of germs; moreover*, *the environment*, *the water we drink*, *bad smells*, *waste everywhere*… *When I find myself scratching my skin constantly and I see that lesions start to appear on my skin*. *I tell myself that antibiotics can make it all disappear*.”

(Male respondent #1)

It was interesting to learn that some female participants commonly used antibiotics during menstruation to prevent infection or when they experienced a burning sensation or tingling in the genital area. Some participants said:

“… *In addition*, *I am a woman*: *when I have my period*, *I feel very bad*, *I have tingling*, *pain… When that happens*, *I disinfect the water with detol (antiseptic) and I take amoxicillin*. *I do not know about the others*, *but this is what I do*. *Every woman has her habits*.”

(Female respondent #7)

“*During my period*, *I take antibiotics to prevent infections… I don’t do it like the others who take them every month*. *I do this randomly after a month or two*. *It depends… It’s by intuition… I learned this from my sister-in-law*, *the wife of my big brother*. *She told me it helps get rid of all the "dirt*.*" Since I’m not someone who likes to take medication*, *I’d rather not do it every month*. "

(Female respondent #12)

“… *I use amoxicillin during my period*. *Towards the end of my period*, *I buy a sleeve of amoxicillin and take one pill in the morning and another in the evening… mostly to protect myself*. *You see the environment we live in and on top of that I have very painful times… Here we have shared showers*, *everyone goes there and we all use the same buckets for bathing*. *Good hygiene is not guaranteed*: *Some wash their dirty clothes in these buckets*, *others dip their feet in them*. *That’s why I prefer to protect myself that way*.”

(Female respondent #16)

### Prescription, purchase, and use of antibiotics

Based on participant responses, we identified several sources advising use of antibiotics. These included in order of importance: self-medication or advisement from non-medical family members, pharmacist or pharmacy salesperson, nurses, and doctors.

***Self-medication*** was very common. Several participants mentioned that they relied on their personal medical experience for antibiotic use. The knowledge was based on previous experience (e.g. previous antibiotic prescriptions from health care providers), or their educational training (for those with a medical background). In a few instances, they relied on advice from family members or friends.

“*By going to the hospital with the kids*, *I got a handle on the routine checkups that are ordered and the medications that doctors prescribe*. *Another thing*, *I jealously guard the medical prescriptions and use when I need need them*, *especially when I don’t have the money*. *When I encounter the same problem*, *I buy the same medicines and it works*.”

(Female respondent #16)

“*I am not a health professional but for several years I ran a pharmacy*. *When people came to buy products for this or that pathology*, *I often remembered what the doctors prescribed*. *In addition*, *I have often fallen ill*: *even now I am sick but I just cannot afford to go to the hospital*. *I have a fever*, *my eyes hurt*,*… I’m just going to use my knowledge to buy products in pharmacies*.”

(Female respondent #9)

Other participants sought advice from pharmacists or pharmacy outlet salespersons for treatment of symptoms or for alternative treatments when the treatments initiated at home did not work.

“*I don’t waste my money in the hospital*. *For frequent pathologies such as malaria and typhoid fever I refer to old prescriptions or I discuss with the pharmacy salesperson*.”

(Female respondent #8)

“… *There is a gentleman who ran a drugstore not far from here*, *who had a reputation of being a good prescriber*… "

(Female respondent #2)

Nurses were also cited as important players in the prescription of antibiotics in the community. Two of the three nurses included in this study reported having prescribed antibiotics to people who came to seek advice from them. One of them said: “*I trust antibiotics… especially those in the beta lactam group and amoxicillin… they are antibiotics that I use a lot… because I trust them… when I use them*, *I have good results in my patients and even those I treat at home when they are sick*.” (Male respondent #1)

For some participants, visiting a doctor was routine when feeling unwell, through for many it was the last resort when other solutions did not resolve the problem.

The lack of financial means was the most commonly reported reason for not visiting a health facility when sick. Most participants mentioned that they could not afford the cost of going to the hospital every time a health problem arose. Therefore, they relied on self-medication, relatives, or healthcare providers outside of the health care setting.

“*The hospital is very expensive and we don’t have enough money*. *To treat a pathology like malaria in a local clinic you have to spend up to 80 US dollars while with 10 dollars you can buy Lutter Injectable (antimalarial drug) and have the same results*."

(Male respondent #10)

“*The hospital is the ultimate level*. *When you feel that your body is not doing well despite everything you’ve taken*, *you have to go to the hospital to find out more*.”

(Female respondent #16)

“*… Due to lack of money many do not even buy the full box of medicine*. *They buy half of the tablets and they stop using them as soon as they feel better*.*"*

(Female respondent #4)

In addition to financial barriers, beliefs such as going to the hospital for the slightest health problem would attract a spirit of sickness were important barriers to visiting the hospital for health care. One participant said: “*I believe that in the hospital there are evil spirits that aggravate the disease and cause people to spend a lot money unnecessarily*.” (Female respondent #8)

### Dosage and duration of treatment

Many participants relied on the advice of prescribers for the dosage and duration of treatment. Those who self-medicated relied on the package leaflet for the dosage and duration of treatment, while others referred to past medical prescriptions: “*Before I used to give it [to my child] once a day*, *but I noticed that when I go to the hospital for more serious cases*, *they always give it 3 times a day*. *So I too started giving it 3 times a day…*” (Female respondent #2)

Age, weight and severity of illness were also cited as factors that would determine the dosage and duration of treatment: “*By the way*, *you have to take into account the age*, *the weight*,*… you have to consider the general condition of the person*. *For example*, *by observing a person of a certain age*, *we can tell ourselves that two amoxicillin tablets are going to be too much for them and therefore we should first give one and see how they progress*…” (Female respondent #1)

“*I give according to age*. *Under 4 years old*, *I give half of a 500 mg tablet in the morning and the other half at night*. *If I happen to miss the morning treatment*, *I give one 500 mg tablet altogether to make up for the morning dose*.”

(Female respondent #3)

Very often, the stopping of the treatment was contingent upon the cessation of symptoms: “*I give one tablet in the morning and another in the evening until the children stop coughing and then I stop*.” (Female respondent #14)

“*Usually when I buy a box (of medicine) I finish it*. *Usually 12 tablets*. *If there is no improvement*, *I will take a second box*.”

(Male respondent #10)

### Storage of antibiotics in the household

We asked participants to show us where they stored their medicines and to show a sample of available medicines in the household to assess medication storage. Storage of antibiotics (including leftover antibiotics) and other medicines in the household was a common practice among the participants. Many participants lived in makeshift houses made of sheet metal, a material that conducts heat and humidity, particularly in a country where the temperature is hot year round. Some antibiotics observed were altered by humidity and had questionable coloring. A few participants kept their medication in a dry and clean place, while others did not store medication at home as could not afford to purchase the full course of treatment due to limited financial means.

### Antibiotic risks

There was limited antibiotic risk knowledge among the participants. Many were not aware of the risks associated with antibiotic use, while others mentioned that antibiotics could be harmful but were not able to elaborate on what the risks were. Of all the participants, only four people were familiar with the term “antibacterial resistance.” Participants were more aware of the benefits of antibiotics.

Some participants mentioned the following:

"… *I’d like to know the real risks of antibiotics*. *What happens when you don’t finish your treatment*. *I myself often stop my treatment due to negligence*.”

(Female respondent #3)

“*When you take antibiotics too frequently*, *that is to say that for the slightest discomfort you resort to antibiotics*, *in the end a resistance is created to this antibiotic which ends up losing its effectiveness on the person*.”

(Female respondent #11)

During the interviews, participants told us about possibly signs of antibiotic resistance though they did not necessarily recognize it as such. One participant said: “… *when I’m sick*, *I don’t take amoxicillin… it doesn’t work for me*. *When I take it*, *there is no effect*. *I prefer Ciprofloxacin or Clamoxyl or even penicillin tablets*: *these have an effect on me… I was taking amoxicillin and it was working well except at one point I started to notice that it didn’t work as well as before*. *I have stopped taking it and I am only giving it to the children*.” (Male respondent #13)

### Perceived quality of antibiotics available in the market

Participants were concerned about the quality of the medicines available in the market. Some had experience with purchasing antibiotics with tampered expiry dates or having used medicines that they perceived to be less effective due to low concentration of the active pharmaceutical ingredient.

One participant said:

"… *my husband once bought expired medicine… They changed the expiration date on the packaging and upon opening the box the medicine had already changed color*. *It was when I checked the box that I noticed it was an expired product*. "

(Female respondent #11)

### Source of information

Participants were aware of their limited knowledge in health and the lack of financial means to go to the hospital and sought “good information” from health professionals, particularly nurses and salespeople at pharmacy outlets. However, the information or care they received were not always appropriate. Some participants said:

“…… *My sister ran into a nurse who gave her a double dose of Norfen (norfloxacin)*. *We stopped the treatment once we found out [that it was wrong]*, *but she has gastritis to this day*.”

(Male respondent #10)

“*The people who sell in the pharmacies are not always pharmacists or health professionals*…”

(Female respondent #11)

## Discussion

This is the first qualitative study to investigate community knowledge of antibiotics and practices regarding antibiotic use in Kinshasa, DRC. Overall, this study showed that the inappropriate use of antibiotics is very common and is a result of an interplay of multiple factors including limited knowledge of antibiotic risks and indications, easier access to antibiotics, self-medication of non-prescribed medicines or prescriptions made by unqualified people (e.g. relatives, pharmacy outlet salespersons), participant perceptions of their high risk for infections (due to living conditions and unsanitary environment), and financial barriers to accessing appropriate health care.

Although most participants in this study were familiar with the term “antibiotic”, they had limited knowledge with regards to the indications and the risks associated with the use of antibiotics. The ineffectiveness of antibiotics against viral infection was not common knowledge among the participants. The reported indications for which they used antibiotics (e.g. cough; fever; etc.) were not justified; considering for example that “cough” is a symptom of conditions and can be due to viral infection which is not sensitive to antibiotics [26]. In addition, cough and fever in a tuberculosis endemic zone could delay proper diagnosis and treatment of tuberculosis [26, 27]. Although prescription of antibiotics is a medical procedure, we observed that, for our study population, the antibiotic prescriber is determined by the sector the patient uses first. In general, participants relied on their personal knowledge or on the knowledge of those around them (self-medication or advice from relatives and friends) before turning to health professionals of any qualification. They had limited knowledge on the dosage of antibiotics and the duration of the full course of treatment which often was determined by improvement of symptoms. In the DRC, while the law prohibits non-prescription over-the-counter sales of antibiotics, this law has been poorly enforced. This has created an environment promoting self-medication practices [22]. A previous study in the DRC documented a self-medication prevalence of 59.6%, with antimicrobial drugs accounting for 32.9% of self-medicated drugs [28]. The retail pharmaceutical sector is largely dominated by unregistered drug shops which are generally not run by fully qualified pharmacists [29]. This situation further compounds the risk of inappropriate prescription of antibiotics in the country. The current law regulating pharmaceutical practices in the DRC dates back to 1933 and has not been supported by accompanying regulations that clearly outline the framework of drug prescription in the country [22]. Thus, there is critical need to update the current law with new regulations and also to ensure mechanisms for enforcement of the law and regulations on prescriptions and retail sales of antibiotics in DRC. Previous research also found that self-medication with non-prescribed drugs is a wide-spread practice in many countries in sub-Saharan Africa, one which is fueled by easier access to antibiotics without medical prescription [12, 20, 26, 28, 30–35]. For example, in a recent survey from Ghana, 64.3% of antibiotics were used without prescription (489 out of 761 antibiotic-use episodes) [20]. Horumpende et al. found a high proportion (75%) of non-prescription antibiotic dispensing for cough in Tanzania [26].

The lack of knowledge about antibiotics contributes to their inappropriate use [36, 37]. In Ghana, Afari-Asiedu et al. found that antibiotics were used to treat stomachache and sores on the body [36]. In our study population, awareness about the risks of antibiotics was low and most participants were unfamiliar with the notion that inappropriate use of antibiotics could lead to antibacterial resistance. There was a high perceived risk of infections, which was one of the driving forces for inappropriate use of antibiotics. Particularly, female participants in this study used antibiotics as a prophylaxis to reduce the risk of infection due to the unsanitary conditions of their living environment and other risky practices such as the sharing of toilets and buckets. Interestingly, menstruation was perceived as a vulnerable period for which the use of antibiotics was justified in order to deter the risk of infection. There were other misconceptions such as antibiotics can “get rid of all the dirt,” or relieve menstral pain. It is critical to conduct large-scale quantitative research to assess the extent of such risky practices in the Pakadjuma slum and other similar resource-constrained settings of Kinshasa.

We found that poverty was a significant structural barrier that contributed to the misuse of antibiotics among the participants. The lack of financial resources deterred visits to health facilities and seeking professional treatment was perceived as more expensive than self-medication or seeking medical advice from relatives or salespersons at pharmacy outlets. In the DRC, health care is mostly paid out of pocket (up to 90% of health care expenditure) in both public and private health facilities [23]. In support of our findings, Afari-Asiedu et al. in Ghana found that people who paid for health care without the benefit of health insurance and those who did not seek health care from health centers were over two times more likely to use antibiotics inappropriately [20]. In addition, financial constraints were cited as a factor that influenced community demand of antibiotics in Ghana [38]. In our study, the lack of financial means resulted in the purchase of less than the recommended quantity of medicines just to make the symptoms disappear. In addition, the precarious living conditions which favor sex work and unfavorable hygienic conditions (such as the use of toilets by several households; the exchange of buckets for the shower, etc.) increased the risk of infection, which in turn, led to extensive use of antibiotics among the residents of this community. The role of poverty in the misuse of antibiotics and emergence of antimicrobial resistance has been shown in previous research and reports [39, 40]. A recent qualitative study in rural South Africa has shown that although people have knowledge of where to receive professional healthcare, financial constraints, cultural norms, or habit often guided their choice of whether to actually seek out professional care [41]. In this regard, while community education is an important facet in addressing the inappropriate use of antibiotics, failure to address structural barriers such a poverty and financial insecurity as well as the lack of health insurance and financial risk protection (through universal health coverage) may negatively impact of the benefit of community education [41].

Storage of antibiotics for future use is a common practice in low- and middle-income settings, and it has been shown to be associated with inappropriate use of antibiotics [42, 43]. In a recent study from Angola, 53.92% of the surveyed households (55/102) was found to store antimicrobials [17]. Most participants in our study stored antibiotics. However, the storage conditions did not meet the standards required for storing medicines safely to ensure drug stability (humidity, temperature, light, oxygen, acidity, presence of catalysts and microbial contamination) [44]. The quality of antibiotics available in the marketplace was another theme that emerged from our study. There were accounts of tampering with drug expiry dates or suspected counterfeit antibiotics in the Congolese medicines market. Counterfeit drugs pose a serious threat for the delivery of quality health care. An estimated 40–45% of medicines sold in the DRC are counterfeit according to the “Office Fédéral des Migrations 2014” [45]. Counterfeit and substandard medicines represent 10% of medicines in low-income and middle-income countries [46]. This situation is due limited capacity and infrastructure for regulation, quality control, and law enforcement of drugs in many low- and middle-income countries.

This study has a few limitations. Firstly, the interview guide was developed in French, but the in-depth interviews were conducted in Lingala without a formal procedure and guidelines for translation to ensure accuracy of translation. The interview guide was not pilot tested. However, the principal investigator, fluent in both French and Lingala, using the flexibility of in-depth interviews, ensured that participants understood the topics for discussion. Secondly, it is unclear to what extent the findings of our study can be applied to other similar settings in the DRC. This study has, however, the merit of being the first to explore the inappropriate use of antibiotic use in the household. More research is needed to increase the evidence base regarding inappropriate use of antibiotics in the DRC.

## Conclusion

There is inappropriate use of antibiotics in the Pakadjuma slum, which is fueled by limited knowledge of the risk antibiotic use and indications, perceptions of being at high risk for infections, widespread self-medication behavior, and structural factors such as poverty and weak regulatory systems for quality control of medicines. In light of these findings, interventions for appropriate use of antibiotics should address community education in order to raise awareness on the risks of antibiotics and good practices for antibiotic use, as well as structural barriers including poverty, financial insecurity, and weak regulatory systems for quality control of medicines.

## Supporting information

S1 File Interview guide. (DOCX)

S2 File Information sheet and consent form. (DOCX)

S3 File Qualitative data-analytical transcripts. (DOCX)
