# Supplementary material for: A qualitative study on community use of antibiotics in Kinshasa, Democratic Republic of Congo
Source: PLoS One. 2022 Apr 27;17(4):e0267544. doi: 10.1371/journal.pone.0267544 (PMC9045656; doi:10.1371/journal.pone.0267544)
Supplement: S1 File — (DOCX) [file pone.0267544.s001.docx]

**GUIDE D’ENTRETIEN**

1. **Introduction**

Bonjour Mr (Mme),

Je suis le Dr Aurélie KOHO PUNGU SHEMBO, médecin généraliste et actuellement en dernière année de master en santé publique à l’Université Catholique de Louvain à Bruxelles en Belgique. Le programme académique prévoit que tout étudiant en dernière année rédige un mémoire.

Pour ma part j’ai choisi de travailler sur L’utilisation des antibiotiques dans les ménages du quartier de Pakadjuma. Cette étude a pour objectif de Documenter et comprendre le mode d’acquisition, d’utilisation, de conservation et de gestion des antibiotiques par les usagers à domicile.

1. **Information sur l’entretien**
2. **Pourquoi vous ? :** vous êtes adultes et vous avez la responsabilité de veiller sur vos traitements à domicile ou celui des autres personnes (enfants ou adultes) partageant le même ménage que vous. Vous répondez donc à nos critères et vous détenez certainement les informations que nous recherchons.
3. **Comment avez-vous été sélectionné ? :** Nous somme passé par une personne qui vous connaissez et vous a jugé éligible pour répondre à nos question. C’est cette dernière qui nous à orienter vers vous et à votre tours nous vous prions de nous orienter vers d’autres personnes que vous connaissez et qui peuvent répondre à nos questions.
4. **Déroulement de l’entretien :** pour nous permettre de mieux analyser les données recueillies lors de l’entretien, nous procédons à l’enregistrement de l’entretien. Toutefois vous avez la liberté d’interrompre l’entretien à n’importe quel moment (montrer la touche “stop”). l’entretien ne sera écouté que par moi et probablement mes directeurs de mémoire. Dans tous les cas il ne sera pas possible de vous identifier car votre identité ne sera pas mentionnée.
5. **Consentement** : nous ne pouvons réaliser cet entretien qu'avec votre accord. pour cela il y'a un formulaire de consentement que nous devons lire ensemble, ensuite si vous êtes d’accord pour répondre à nos question vous nous le ferez savoir en signant le formulaire de consentement éclairé.
6. **Avez à ce niveau des questions ou préoccupations ?**
7. **Entretien proprement dit**

- Date :
- Âge :
- Sexe
- Nombre des personnes dans le ménage

**Questions d’ouverture**

- Combien de personnes vivent dans la maison ?
- Est-ce bien vous qui gérer les médicaments pour tout le monde ?
- Parlez-moi de vous (, quel âge ? profession, niveau d’études, responsabilité dans le ménage…)
- dites nous ce qui explique que cette responsabilité vous revienne particulièrement (soin à la maison)

**Questions de transition**

- Que savez-vous des antibiotiques? (rôle, avantages, …)
- Avez-vous une petite pharmacie chez vous? si oui pouvez-vous la montrer? (Photo), pouvez- vous, parmi les médicaments présents dans votre pharmacie nous montrer les antibiotiques?
- Qu’est ce qui vous motive à en faire recours?

**Questions clé**

- Comment vous en procurez-vous ?
- vous est-il déjà arrivé de prendre ou de donner des antibiotiques au regard de certains symptômes ? Si oui :
- sur base de quoi faite vous le choix de l’antibiotique?
- Comment calculez-vous la dose ?
- comment estimez-vous la durée de traitement?
- qu’est-ce qui vous motive à interrompre un traitement?
- d'où vous vient cette expérience?
- Comment conservez-vous les restes ?
- comment vous en débarrassez-vous?
- d’après votre expérience quelle est la différence entre les traitements prescrits à l'hôpital et ceux que vous prenez par vous même à domicile ?
- parlez nous de quelques risques que présentent les antibiotiques

**Question de clôture**

- avez-vous quelque un commentaire à faire sur ce sujet?

1. **CONCLUSION**

Je vous remercie infiniment pour le temps que vous avez consacré à cette interview. Vous avez mes coordonnées et vous pouvez me contacter si vous avez une préoccupation particulière en rapport avec ce sujet.

Durée de l’interview…………….
