# Supplementary material for: A qualitative study on community use of antibiotics in Kinshasa, Democratic Republic of Congo
Source: PLoS One. 2022 Apr 27;17(4):e0267544. doi: 10.1371/journal.pone.0267544 (PMC9045656; doi:10.1371/journal.pone.0267544)
Supplement: S2 File — (DOCX) [file pone.0267544.s002.docx]

**FORMULAIRE D’INFORMATION ET DE CONSENTEMENT**

**Acquisition, l’utilisation et la conservation des antibiotiques dans les ménages des quartiers périphérique de Kinshasa : cas du quartier de Pakadjuma**

*Avant que vous n’acceptiez de participer à cette étude, nous vous invitons à prendre connaissance de ses implications en termes d’organisation, avantages et risques éventuels, afin que vous puissiez prendre une décision en toute connaissance de cause. Ceci s’appelle donner un « consentement éclairé ».*

*Veuillez lire attentivement ces quelques pages d’information et poser toutes les questions que vous souhaitez à l’investigateur.*

**Contextes et responsables**

Cette étude s’inscrit dans le cadre d’un mémoire de fin de master en santé publique du Docteur KOHO PUNGU SHEMBO Aurélie, étudiante à l’Université catholique de Louvain en Belgique. Ce mémoire est dirigé par les Professeurs Olivia Dalleur et Jean Macq, assistés par le Dr Angèle Dohou.

**Si vous avez besoin d’informations complémentaires, mais aussi en cas de problème ou d’inquiétude, vous pouvez nous contacter sur l’adresse:** [**aurelieshembo@gmail.com**](mailto:aurelieshembo@gmail.com) **ou appeler le 00243**

**Financement du projet**

Ce projet ne bénéficie d’aucun financement.

**Si vous participez à cette étude, vous devez savoir que :**

- Cette étude clinique est mise en œuvre après évaluation par un / plusieurs comité(s) d’éthique.
- Votre participation est volontaire et doit rester libre de toute contrainte. Elle nécessite la signature d’un document exprimant votre consentement. Même après l’avoir signé, vous pouvez arrêter de participer en informant l’investigateur.
- Les données recueillies à cette occasion sont confidentielles et votre anonymat est garanti lors de la publication des résultats.
- Vous pouvez toujours contacter l’investigateur ou un membre de son équipe si vous avez besoin d’informations complémentaires.

**Nos objectifs**

Notre recherche vise à découvrir par des interviews avec des habitants du quartier de Pakadjuma à Kinshasa comment ils obtiennent, utilisent et conservent leurs antibiotiques pour usage ménager.

**Ce que ça implique pour vous :**

Vous êtes adulte et vous vous occupez du suivi de votre propre traitement et /ou celui des autres membres de votre ménage. Votre expérience nous intéresse nous permettra de récolter des informations utiles pour notre projet.

Vous participerez à une entrevue d'environ 30 à 45 minutes. Cette entrevue aura lieu dans votre domicile, selon vos disponibilités. Vous aurez à répondre à des questions sur la façon dont vous vous procurez, administrez, gérez et conservez les antibiotiques dans votre ménage. Cette entrevue sera enregistrée sur bande audio et nous prendrons des photos de votre petite pharmacie ménagère. Les informations seront ensuite anonymisées.

**Description des risques et bénéfices**

Aucun risque, en termes de santé, ne peut être lié à votre participation à cette étude.

De même, vous ne devez pas vous attendre à des bénéfices personnels du fait de votre participation à l’étude. Sachez seulement que votre participation nous permettra de mieux comprendre l’utilisation au domicile des antibiotiques et donc de proposer de meilleurs traitements à l’avenir.

**Compensations financières**

Aucune compensation financière n’est prévue. Toutefois le chercheur pourrait vous proposer un petit rafraichissement pour détendre l’atmosphère pendant l'interview.

**Droit de retrait sans préjudice de la participation**

Il est entendu que votre participation à ce projet est tout à fait volontaire et que vous restez libre, à tout moment, de mettre fin à votre participation sans avoir à motiver votre décision ni à subir de préjudice de quelque nature que ce soit. Toutefois, lorsque vous aurez rempli et retourné le questionnaire, il sera impossible de détruire les données puisqu’aucune information permettant d’identifier les répondants n’a été recueillie.

**Confidentialité**

Durant votre participation à ce projet, les données sont recueillies et conservées sous la responsabilité du chercheur responsable. Seuls les renseignements nécessaires à la bonne conduite du projet de recherche seront recueillis et tout sera anonymisé. Ils peuvent comprendre les informations suivantes: sexe, âge, revenu, niveau d’étude, photographies de votre pharmacie à domicile, transcription des enregistrements audio. Nous ne recueillons ni le nom ni les photos ou vidéos de vous. Les enregistrements seront détruits après transcription.

**Surveillance des aspects éthiques et identification du président du Comité d’éthique**

Le Comité national d’éthique de la santé a approuvé ce projet de recherche et en assure le suivi. De plus, il approuvera au préalable toute révision et toute modification apportée au formulaire d’information et de consentement, ainsi qu’au protocole de recherche. Vous pouvez parler de tout problème éthique concernant les conditions dans lesquelles se déroule votre participation à ce projet avec la responsable du projet ou expliquer vos préoccupations à M………………………………………………………… président du Comité d’éthique de la recherche, en communiquant par l’intermédiaire de son secrétariat au numéro suivant: …………………………………..ou par courriel à:.......................................................

**Consentement libre et éclairé du participant**

Je déclare que j’ai été informé sur la nature de l’étude, son but, sa durée, et ce que l’on attend de moi. J’ai pris connaissance du document d’information.

J’ai eu suffisamment de temps pour y réfléchir et en parler avec une personne de mon choix.

J’ai eu l’occasion de poser toutes les questions qui me sont venues à l’esprit et j’ai obtenu une réponse favorable à mes questions.

J’ai compris que des données me concernant seront récoltées pendant toute ma participation à cette étude et le promoteur de l’étude se portent garant de la confidentialité de ces données.

Je consens au traitement de mes données personnelles selon les modalités décrites dans la rubrique traitant de garanties de confidentialité. Je donne également mon accord au transfert et au traitement de ces données dans d’autres pays que la RDC.

J’ai reçu une copie de l’information au participant et du consentement éclairé.

Nom, prénom, date et signature du volontaire.

**Déclaration de responsabilité des chercheurs de l’étude**

Je soussigné,

Confirme avoir fourni oralement les informations nécessaires sur l'étude et avoir fourni un exemplaire du document d’information au participant.

Je confirme qu'aucune pression n'a été exercée pour que le patient accepte de participer à l'étude et que je suis prêt à répondre à toutes les questions supplémentaires, le cas échéant.

Je confirme travailler en accord avec les principes éthiques énoncés dans la « Déclaration

**FORMULAIRE D’INFORMATION ET DE CONSENTEMENT**

**Acquisition, l’utilisation et la conservation des antibiotiques dans les ménages des quartiers périphérique de Kinshasa : cas du quartier de Pakadjuma**

*Avant que vous n’acceptiez de participer à cette étude, nous vous invitons à prendre connaissance de ses implications en termes d’organisation, avantages et risques éventuels, afin que vous puissiez prendre une décision en toute connaissance de cause. Ceci s’appelle donner un « consentement éclairé ».*

*Veuillez lire attentivement ces quelques pages d’information et poser toutes les questions que vous souhaitez à l’investigateur.*

**Contextes et responsables**

Cette étude s’inscrit dans le cadre d’un mémoire de fin de master en santé publique du Docteur KOHO PUNGU SHEMBO Aurélie, étudiante à l’Université catholique de Louvain en Belgique. Ce mémoire est dirigé par les Professeurs Olivia Dalleur et Jean Macq, assistés par le Dr Angèle Dohou.

**Si vous avez besoin d’informations complémentaires, mais aussi en cas de problème ou d’inquiétude, vous pouvez nous contacter sur l’adresse:** [**aurelieshembo@gmail.com**](mailto:aurelieshembo@gmail.com) **ou appeler le 00243**

**Financement du projet**

Ce projet ne bénéficie d’aucun financement.

**Si vous participez à cette étude, vous devez savoir que :**

- Cette étude clinique est mise en œuvre après évaluation par un / plusieurs comité(s) d’éthique.
- Votre participation est volontaire et doit rester libre de toute contrainte. Elle nécessite la signature d’un document exprimant votre consentement. Même après l’avoir signé, vous pouvez arrêter de participer en informant l’investigateur.
- Les données recueillies à cette occasion sont confidentielles et votre anonymat est garanti lors de la publication des résultats.
- Vous pouvez toujours contacter l’investigateur ou un membre de son équipe si vous avez besoin d’informations complémentaires.

**Nos objectifs**

Notre recherche vise à découvrir par des interviews avec des habitants du quartier de Pakadjuma à Kinshasa comment ils obtiennent, utilisent et conservent leurs antibiotiques pour usage ménager.

**Ce que ça implique pour vous :**

Vous êtes adulte et vous vous occupez du suivi de votre propre traitement et /ou celui des autres membres de votre ménage. Votre expérience nous intéresse nous permettra de récolter des informations utiles pour notre projet.

Vous participerez à une entrevue d'environ 30 à 45 minutes. Cette entrevue aura lieu dans votre domicile, selon vos disponibilités. Vous aurez à répondre à des questions sur la façon dont vous vous procurez, administrez, gérez et conservez les antibiotiques dans votre ménage. Cette entrevue sera enregistrée sur bande audio et nous prendrons des photos de votre petite pharmacie ménagère. Les informations seront ensuite anonymisées.

**Description des risques et bénéfices**

Aucun risque, en termes de santé, ne peut être lié à votre participation à cette étude.

De même, vous ne devez pas vous attendre à des bénéfices personnels du fait de votre participation à l’étude. Sachez seulement que votre participation nous permettra de mieux comprendre l’utilisation au domicile des antibiotiques et donc de proposer de meilleurs traitements à l’avenir.

**Compensations financières**

Aucune compensation financière n’est prévue. Toutefois le chercheur pourrait vous proposer un petit rafraichissement pour détendre l’atmosphère pendant l'interview.

**Droit de retrait sans préjudice de la participation**

Il est entendu que votre participation à ce projet est tout à fait volontaire et que vous restez libre, à tout moment, de mettre fin à votre participation sans avoir à motiver votre décision ni à subir de préjudice de quelque nature que ce soit. Toutefois, lorsque vous aurez rempli et retourné le questionnaire, il sera impossible de détruire les données puisqu’aucune information permettant d’identifier les répondants n’a été recueillie.

**Confidentialité**

Durant votre participation à ce projet, les données sont recueillies et conservées sous la responsabilité du chercheur responsable. Seuls les renseignements nécessaires à la bonne conduite du projet de recherche seront recueillis et tout sera anonymisé. Ils peuvent comprendre les informations suivantes: sexe, âge, revenu, niveau d’étude, photographies de votre pharmacie à domicile, transcription des enregistrements audio. Nous ne recueillons ni le nom ni les photos ou vidéos de vous. Les enregistrements seront détruits après transcription.

**Surveillance des aspects éthiques et identification du président du Comité d’éthique**

Le Comité national d’éthique de la santé a approuvé ce projet de recherche et en assure le suivi. De plus, il approuvera au préalable toute révision et toute modification apportée au formulaire d’information et de consentement, ainsi qu’au protocole de recherche. Vous pouvez parler de tout problème éthique concernant les conditions dans lesquelles se déroule votre participation à ce projet avec la responsable du projet ou expliquer vos préoccupations à M………………………………………………………… président du Comité d’éthique de la recherche, en communiquant par l’intermédiaire de son secrétariat au numéro suivant: …………………………………..ou par courriel à:.......................................................

**Consentement libre et éclairé du participant**

Je déclare que j’ai été informé sur la nature de l’étude, son but, sa durée, et ce que l’on attend de moi. J’ai pris connaissance du document d’information.

J’ai eu suffisamment de temps pour y réfléchir et en parler avec une personne de mon choix.

J’ai eu l’occasion de poser toutes les questions qui me sont venues à l’esprit et j’ai obtenu une réponse favorable à mes questions.

J’ai compris que des données me concernant seront récoltées pendant toute ma participation à cette étude et le promoteur de l’étude se portent garant de la confidentialité de ces données.

Je consens au traitement de mes données personnelles selon les modalités décrites dans la rubrique traitant de garanties de confidentialité. Je donne également mon accord au transfert et au traitement de ces données dans d’autres pays que la RDC.

J’ai reçu une copie de l’information au participant et du consentement éclairé.

Nom, prénom, date et signature du volontaire.

**Déclaration de responsabilité des chercheurs de l’étude**

Je soussigné,

Confirme avoir fourni oralement les informations nécessaires sur l'étude et avoir fourni un exemplaire du document d’information au participant.

Je confirme qu'aucune pression n'a été exercée pour que le patient accepte de participer à l'étude et que je suis prêt à répondre à toutes les questions supplémentaires, le cas échéant.

Je confirme travailler en accord avec les principes éthiques énoncés dans la « Déclaration d’Helsinki».

Nom, Prénom, Date et signature du chercheur responsable
