# Supplementary material for: A qualitative study on community use of antibiotics in Kinshasa, Democratic Republic of Congo
Source: PLoS One. 2022 Apr 27;17(4):e0267544. doi: 10.1371/journal.pone.0267544 (PMC9045656; doi:10.1371/journal.pone.0267544)
Supplement: S3 File — (DOCX) [file pone.0267544.s003.docx]

Qualitative data of the analytical transcripts

|  |  |
| --- | --- |
| **Categories** | **Selected quotes from the participants** |
| **Knowledge, indications, and use of antibiotics** | *“Antibiotics are medicines… Medicines that treat diseases… for example amoxicillins they are antibiotics are medicines that treat infections, which I know it can help for example when you cough, you can combine it with cough suppressants me for example I use a lot of antibiotics when I have a fever I have to take antibiotics but the antibiotic that I find effective... often I use amoxi, cifin that's what I use.”* (Female respondent #6)  *“These are medicines that are given to cure illnesses.”* (Female respondent #5)  “*Antibiotics are medicines that treat infections. There are also antibiotics that we use to clean surfaces… These are drugs like ammoxi, metronidazole and others.*” (Female respondent #7)  “*These are drugs that treat infections like amoxicillin, gentamicin, erythromycin…these are the antibiotics that I take a lot.”* (Female respondent #9)  “*Antibiotics are the drugs that are taken to raise the level of antibodies in the body to fight diseases, for example there is amoxicillin, shalcip, norfen augmentin, perdolan... these are the ones I use*” (Male respondent #10)  “*I hear about antibiotics but to be honest I don't know the correct definition. I know it cures germs, pain*” (Female respondent #13)  “*Antibiotics are medicines that treat diseases. Amoxi, for example, are antibiotics.*” (Female respondent #15)  “*There are tranquilizers, painkillers*” (Female respondent #15)  “*Antibiotics are pills or capsules that treat disease. That's what I know about antibiotics. I can show you some drugs I have here (she shows us some drugs including amoxicillin and gentamicin which are antibiotics)*” (Female respondent #17)  “*Just looking at the environment we live in, you have to know that there are a lot of germs; moreover, the environment, the water we drink, bad smells, waste everywhere ... When I find myself scratching my skin constantly and I see that lesions start to appear on my skin. I tell myself that antibiotics can make it all disappear* .” (Male respondent #1)  *“When my wife has white discharge or if she tickles her private parts I give her antibiotics.”* (Male respondent #1)  “*When my children have skin infections, when I notice funny pimples on the skin I give antibiotics. Sometimes in case of malaria accompanied by influenza*” (Female respondent #2)  “... *In addition, I am a woman: when I have my period, I feel very bad, I have tingling, pain… When that happens, I disinfect the water with detol (antiseptic) and I take amoxicillin. I do not know about the others, but this is what I do. Every woman has her habits.*” (Female respondent #7)  *“First, the choice of antibiotic depends on its sensitivity to the microbe. If you do not get examined how would you know which germ it is and which antibiotic is sensitive to this germ”.* *“ (Female respondent #6)*  “*During my period, I take antibiotics to prevent infections ... I don't do it like the others who take them every month. I do this randomly after a month or two. It depends ... It's by intuition ... I learned this from my sister-in-law, the wife of my big brother. She told me it helps get rid of all the "dirt." Since I'm not someone who likes to take medication, I'd rather not do it every month.* " (Female respondent #12)  *“These are medicines that treat infections such as typhoid. For example there my son has pimples on the skin that can hide a skin infection and justify taking antibiotics… I give more clamoxyl; Also when I have malaria, I prefer to combine the antimalarial with an antibiotic to treat other possible infections at the same time. (Female respondent #12)*  “... *I use amoxicillin during my period. Towards the end of my period, I buy a sleeve of amoxicillin and take one pill in the morning and another in the evening ... mostly to protect myself. You see the environment we live in and on top of that I have very painful times… Here we have shared showers, everyone goes there and we all use the same buckets for bathing. Good hygiene is not guaranteed*: *Some wash their dirty clothes in these buckets, others dip their feet in them. That's why I prefer to protect myself that way* .” (Female respondent #16)  *“What you should know first is that my pakadjuma neighborhood is a neighborhood where people who do not have enough means live. So they resort to cheaper drugs? I tell you this because I run a pharmacy and the most purchased antibiotics are amoxicillin, ceftriaxone then ciprofloxacin and cefixime… these are drugs that fight infections in the body” (Male respondent #4)*  “*The indication depends on the pathology and each pathology is expressed by symptoms. It is when we are oriented by the symptoms that it can be typhoid fever or malaria that we can know what to prescribe. From experience I know how to recognize certain symptoms and this is what allows me to prescribe medication*” *” (Male respondent #4)*  *“When a person has recurrent fevers despite taking antipyretics or in the event of chills lasting more than 3 days, antibiotics can already be given”.* *(Male respondent #4)*  *“..tingling of the skin or private parts, fungal skin infection, painful periods… because I really have a problem with painful periods. These are the antibiotics I take for pain relief.”* *(Female respondent #9)*  “*My son has asthma, I tried several times to treat him with traditional medicine but it didn't work. One day I met a gentleman whose son had the same problem and he advised me to give antibiotics and since then the child has not had seizures for 3 months*.” *(Female respondent #18)*  *“It depends. Like there for example I have an abscess at the very beginning I had taken cefotaxime that my child was using, when I saw that it didn't really work, I took another medicine ( muscle Plus) and it didn't work as well; that's how I paid for clamoxyl that's really helped to soften the abscess and cure me well. “ (Female respondent #6)* |
| **Prescription, purchase, and use of antibiotics** | *“Because I often go to the hospital, I have become knowledgeable of the medical exam and medicines that doctors prescribe. Another thing, I jealously keep the medical prescriptions at home to use them when needed, especially when I have no money. In general when it is the same problems I buy the same products and it works. (Female respondent #6)*  *“In Kinshasa when you go to the hospital the diagnoses are the same: malaria, typhoid... and it's always the same treatment. So when the same symptoms come back, we give the same treatment.” (Female respondent #7)*  “*By going to the hospital with the kids, I got a handle on the routine checkups that are ordered and the medications that doctors prescribe. Another thing, I jealously guard the medical prescriptions and use when I need need them, especially when I don't have the money. When I encounter the same problem, I buy the same medicines and it works.*” (Female respondent #16)  *“At my house, we don't take medicines any how”. I am a heart problem and I don’t take medicines without consulting a doctor. After 3 days of fever or malaise I go to the hospital. Antibiotics are products that can not be taken anyhow. Even for my children I never give medicines anyhow. I always consult a pediatrician because we do not joke with the health of children. Apart from paracetamol, I never give products to children without consulting them”. (Female respondent #11)*  “*I am not a health professional but for several years I ran a pharmacy. When people came to buy products for this or that pathology, I often remembered what the doctors prescribed. In addition, I have often fallen ill: even now I am sick but I just cannot afford to go to the hospital. I have a fever, my eyes hurt, ... I'm just going to use my knowledge to buy products in pharmacies.*” (Female respondent #9)  *“The way antibiotics are given in this country is truly deplorable. Before, people went to the hospital as a priority, but now this is no longer the case. People self-prescribe drugs. For my part, I always go to the hospital and when I have no money I take advice from a pharmacist.”* (Female respondent #5)  “*I have always noticed that just going to buy medicines in a pharmacy does not cure children in the long term. Often there are several relapses. So I prefer to go see the doctor so that he can consult the child properly and treat him well once and for all. In our circles there are a lot of fake nurses and fake pharmacists, you have to be careful*” (Female respondent #13)  “*During all these years I took my children to the hospital and by habit I ended up acquiring experience with certain recurring diseases and with the products that doctors usually prescribe.”* (Female respondent #15)  “*Often it is at the pharmacy that I am prescribed. I only go to the hospital if what I gave didn't work. They often tell me to only come late, but I have no choice*.” (Female respondent #17)  “*I don't waste my money in the hospital. For frequent pathologies such as malaria and typhoid fever I refer to old prescriptions or I discuss with the pharmacy salesperson.*” (Female respondent #8)  “… *There is a gentleman who ran a drugstore not far from here, who had a reputation of being a good prescriber* ... " (Female respondent #2)  *“I make sure I go to a trusted pharmacy outlet run by someone I can trust”* *(Female respondent #3)*  *“I sometimes give advice to my friends. For example, when a friend's child falls ill and has symptoms similar to those my child had and for which the doctors prescribed a certain treatment that worked, I can recommend the same treatment to my friend to avoid consultation fees”. (Female respondent #3)*  *“It has happened to me, for lack of money, to be consulted for free by my neighbor who is a nurse…” (Female respondent #12)*  “*I trust antibiotics… especially those in the beta lactam group and amoxicillin… they are antibiotics that I use a lot… because I trust them… when I use them, I have good results in my patients and even those I treat at home when they are sick* .” (Male respondent #1)  “*The hospital is very expensive and we don't have enough money. To treat a pathology like malaria in a local clinic you have to spend up to 80 US dollars while with 10 dollars you can buy Lutter Injectable (antimalarial drug) and have the same results.*" (Male respondent #10)  “*The hospital is the ultimate level. When you feel that your body is not doing well despite everything you’ve taken, you have to go to the hospital to find out more*.” (Female respondent #16)  “*I believe that in the hospital there are evil spirits that aggravate the disease and cause people to spend a lot money unnecessarily.*” (Female respondent #8) |
| **Dosage and duration of treatment** | “*Before I used to give it [to my child] once a day, but I noticed that when I go to the hospital for more serious cases, they always give it 3 times a day. So I too started giving it 3 times a day ...* ” (Female respondent #2)  *“I look at the medicine notice. Everything is explained there” (Male respondent #10)*  *“When I buy a pack of 10 tablets I give the whole pack. (Female respondent #6))*  “*By the way, you have to take into account the age, the weight,... you have to consider the general condition of the person. For example, by observing a person of a certain age, we can tell ourselves that two amoxicillin tablets are going to be too much for them and therefore we should first give one and see how they progress* ... ” (Male respondent #1)  “*I give according to age. Under 4 years old, I give half of a 500 mg tablet in the morning and the other half at night. If I happen to miss the morning treatment, I give one 500 mg tablet altogether to make up for the morning dose*. ” (Female respondent #3)  “*I give one tablet in the morning and another in the evening until the children stop coughing and then I stop.*” (Female respondent #14)  “*In general for antibiotics it is at least 5 days of treatment. If the infection is serious, we can give 3 times a day and go up to 7 days of treatment.*” (Male respondent #4)  “*Usually when I buy a box (of medicine) I finish it. Usually 12 tablets. If there is no improvement, I will take a second box.* ” (Male respondent #10)  *“I have difficulty finishing cures and even when it comes to children I stop as soon as I notice that the child is better, that he starts playing again... When I was a teenager, I pretended to drink the products but in reality I threw them away and now that I am grown up, I do not respect the cure, even that of children” . (Female respondent #7)*  “*I take two amoxicillin pills in the morning and two in the evening for the entire menstrual period. Sometimes I start the dose a few days before my period starts and during*” *(Female respondent #13)*  “*Sometimes a week, sometimes 5 days It depends. For menstruation it is during the duration of the rules. As soon as it stops, we stop taking products.*” *(Female respondent #15)*  *“The duration and dose of what the doctor prescribes. If you estimate for yourself you can also be wrong. To each his own. It is better to go to a doctor to follow what the doctors say because we do not joke with health… When I have no money, I do it on credit. They knew that I am a serious person who always honors his debts and therefore they do not refuse me care even if I have nothing in my pocket.”* *(Female respondent #5)*  “*When I go to buy at the pharmacy, I ask the pharmacist about the dose and duration…sometimes I give it to the child until the symptoms disappear and then I stop*.” *(Female respondent #17)* |
| **Antibiotic risks** | "... *I'd like to know the real risks of antibiotics. What happens when you don't finish your treatment. I myself often stop my treatment due to negligence.* ” (Female respondent #3)  “*Myself I had skin problems and I took the same antibiotics as my children but unfortunately it did not work. I went to see a doctor who prescribed me another antibiotic which also did not work. It was when I went back to the same doctor that he referred me to a dermatologist. The latter prescribed me antibiotics combined with skin antiseptics. That's what helped me. The dermatologist had also criticized me for the abuse taking of medication because sometimes we use unsuitable antibiotics or even by habit of using the same thing it risks losing its ability to act on us.*” X2  *“The diarrhoea, the worsening of the disease, uh… the doctor explained taking antibiotics yourself at home is often not adapted to the real needs and can cause more harms than good”* (Female respondent #2)  “*Drug resistance exists. I had a case of a person who suffered from typhoid fever and who was given antibiotics but it did not heal even though these antibiotics respond well to this pathology. We eventually understood that the person had developed resistance to these antibiotics. Another cause of resistance is the poor quality of drugs sold by Indian pharmaceutical companies in this country. For lack of regulation and control, we are sold anything*”  (Female respondent #4)  “*The danger is more related to the dose. As I told you earlier, when I take 2 amoxicillin tablets at the same time, I feel very bad. That means I can't go beyond one tablet. Everyone has a dose that their body supports and beyond which their body can react badly*.” (Female respondent #9)  *“ To tell you the truth, I don't know the risks that antibiotics represent. (Female respondent #12)*  “*I have never heard of the dangers of antibiotics and I don't know of any. If you know them it would be better to tell us about them*”*. (Female respondent #17)*  *“Yes. Often it occurs when you take a lot of antibiotics and the body gets used to these products which become ineffective. In this case, it is better to change antibiotics and take those that are stronger. This has happened to me before: when I took locally made ampicillin it didn't work like it used to. So I opted for Clamoxyl which I currently find more consistent”* (Male respondent #10)  “*When you take antibiotics too frequently, that is to say that for the slightest discomfort you resort to antibiotics, in the end a resistance is created to this antibiotic which ends up losing its effectiveness on the person.* ” (Female respondent #11)  “... *when I'm sick, I don't take amoxicillin… it doesn't work for me. When I take it, there is no effect. I prefer Ciprofloxacin or Clamoxyl or even penicillin tablets: these have an effect on me ... I was taking amoxicillin and it was working well except at one point I started to notice that it didn't work as well as before. I have stopped taking it and I am only giving it to the children*.” (Male respondent #13)  “*We are often told that we must avoid excessive antibiotics and that it is better to go to the hospital, but we do not often follow this advice because we often do not have a choice. We can't afford to go to the hospital all the time.*” (Female respondent #16) |
| **Perceived quality of antibiotics available in the market** | "... *my husband once bought expired medicine ... They changed the expiration date on the packaging and upon opening the box the medicine had already changed color. It was when I checked the box that I noticed it was an expired product*. " (Female respondent #11) |
| **Source of information** | “...*... My sister ran into a nurse who gave her a double dose of Norfen (norfloxacin). We stopped the treatment once we found out [that it was wrong], but she has gastritis to this day.* ” (Male respondent #10)  “*The people who sell in the pharmacies are not always pharmacists or health professionals*...” (Female respondent #11) |
| **Storage** | *“Oh! to be honest, I don't put them in the trash. I leave them in a neglected corner and forget. The next time I need I buy again”.* (Female respondent #3)  *“There is the expiry date on the box, I can keep until the expiry date. But in any case when it comes to syrups I throw away. Once I opened the bottle; after 7 days I throw in the trash.”* (Female respondent #11)  “*I only keep the medicines in capsules but those in bulk I do not keep them. I throw them in the trash because after a certain period of time they start to melt and stick to the paper envelope*” (Female respondent #9)  “*I jealously keep them in a dry box and reuse them as needed. But I don't keep expired drugs*.” (Male respondent #10)  “*I do not often keep antibiotic products because often I buy according to the prescribed dose and I finish it. Next time when I need it, I will buy from Pharmacy again*.”  (Female respondent #13)  “*I only keep fever medication. The rest I throw away or give to other people because keeping medicine at home can invite illness. Sometimes I even throw them in the trash*” (Female respondent #16) |
| **Barriers** | *“I hear that children have to be taken to the hospital because at home they sometimes give uneeded treatments that can calm germs without curing them. This causes many deaths among children. But it is for lack of means that we do this. Lately my daughter was very sick: she had high fevers and had breathing difficulties. I went to the hospital and the doctor asked to carry out 7 laboratory tests. I had no money...I couldn't do all these exams...” (Female respondent #12)*  *“For my case it is complicated because I no longer live with the father of my children, he is irresponsible. Even when the children are sick we cannot count on his help, he gives nothing. I take care of it alone. So it is difficult to take them to the hospital all the time. You have to pay for the consultations, the sheets, the lab exams and then the drugs, it's too much. Last time my son was sick and I went to see a pediatrician. as soon as I paid for the consultation and the file, I didn't have much left for the rest. I finally went to seek advice from my sister who gave me ibumol; This is what I gave the child for 3 days. This is often our* reality. We have the will to go to the hospital but not the means…”*(Female respondent #12)*  “*... Due to lack of money many do not even buy the full box of medicine. They buy half of the tablets and they stop using them as soon as they feel better."* (Female respondent #4)  *“We know that Indian medicines are often not very effective. These Indians are just businessmen who have no concern for the Congolese population. Unfortunately their products are less expensive and the population is using them...currently we are noticing a change in chloramphenicol which is also an antibiotic. Its coloring is no longer the same as before and it seems less effective to us. Maybe all that would also be the basis of his resistance”. (Female respondent #4)*  *“What I can say is that people need to be consulted and prescribed by doctors... I don't always go the hospital due to the lack of money, sometimes I don't have anything . He consults me and I pay later when I have money. I take the medicines in the pharmacy run by his wife. These are people who trust me because I always pay my debts”. (Female respondent #5)*  *“You know especially here in Kinshasa, it is difficult to change things because self-medication is very ingrained in people's heads, especially housewives. People need to be made aware of avoiding self-medication, of going for a consultation when there is a problem. It is better to go even to a small center in the area than to self-medicate at home, it is not good.” (Female respondent #11)*  *“I would like to propose something even if I am only a drop in the ocean: the government must make efforts to better control this (the control of drugs sold on the market). It is really sad in this country... We must also improve the living conditions of the populations because as long as they remain poor, there will be no great impact. The two must go together. On the one hand the awareness of the populations, on the other hand the improvement of their living conditions and added to that the control of the pharmaceutical companies” .” (Female respondent #4)*  *“Patients must be made aware of compliance with the cures prescribed to them. Especially young girls. I would be very happy to see the population trained on the importance and risks of antibiotics.” (Female respondent #1)*  “… I would like to know the real risks of antibiotics. What can happen when you don't finish your cure. Myself speaking to you, it happens that by negligence I abandon a treatment” *(Female respondent #3)*  *“It's really good to talk to us about these things so that we put more seriousness in our way of acting...I think that women must be educated in this matter because men are always gone and it's are we the ones who take care of the treatments of the children at home”* (Female respondent #7) |
